# Landscape analysis of health technology assessment (HTA): systems and practices in Asia

**DOI:** 10.1017/S0266462319000667

**Published:** 2019-06-09

**Authors:** Yot Teerawattananon, Waranya Rattanavipapong, Lydia Wenxin Lin, Saudamini Vishwanath Dabak, Brent Gibbons, Wanrudee Isaranuwatchai, Kai Yee Toh, Boon Piang Cher, Fiona Pearce, Diana Beatriz S Bayani, Ryota Nakamura, Raoh-Fang Pwu, Asrul Akmal Shafie, Deepika Adhikari, Shankar Prinja, Wendy Babidge

**Affiliations:** 1 The Health Intervention and Technology Assessment Program, Ministry of Public Health, Nonthaburi, Thailand; 2 Saw Swee Hock School of Public Health, National University of Singapore, Singapore, Singapore; 3 St. Michael’s Hospital, Li Ka Shing Knowledge Institute, Centre for Excellence in Economic Analysis Research, Toronto, Ontario, Canada; 4 Agency for Care Effectiveness, Ministry of Health, Singapore, Singapore; 5 Health Technology Assessment Unit, Department of Health, Manila, Republic of the Philippines; 6 Hitotsubashi Institute for Advanced Study, Hitotsubashi University, Tokyo, Japan; 7 National Hepatitis C Program Office, Ministry of Health and Welfare, Taipei, Taiwan (R.O.C.); 8 Discipline of Social & Administrative Pharmacy, Universiti Sains Malaysia, Penang, Malaysia; 9 Essential Medicines and Technology Division, Department of Medical Services, Ministry of Health, Thimphu, Bhutan; 10 Department of Community Medicine and School of Public Health, Postgraduate Institute of Medical Education and Research, Chandigarh, India and; 11 Royal Australasian College of Surgeons and University of Adelaide Discipline of Surgery, Adelaide, South Australia, Australia

**Keywords:** Health economics/economic evaluation, Health services/systems research

## Abstract

This paper explores the characteristics of health technology assessment (HTA) systems and practices in Asia. Representatives from nine countries were surveyed to understand each step of the HTA pathway. The analysis finds that although there are similarities in the processes of HTA and its application to inform decision making, there is variation in the number of topics assessed and the stakeholders involved in each step of the process. There is limited availability of resources and technical capacity and countries adopt different means to overcome these challenges by accepting industry submissions or adapting findings from other regions. Inclusion of stakeholders in the process of selecting topics, generating evidence, and making funding recommendations is critical to ensure relevance of HTA to country priorities. Lessons from this analysis may be instructive to other countries implementing HTA processes and inform future research on the feasibility of implementing a harmonized HTA system in the region.

Health technology assessment (HTA) has grown considerably in Asia over the last two decades, with widespread adoption and use of HTA in priority setting for health policy (1–5), particularly among low- and middle-income countries in the region (4). The establishment of the HTAsiaLink network in 2011 has been catalytic in driving this growth sixteen member countries (6). Since 2012, an annual HTAsiaLink conference has brought together members along with global and regional HTA experts to discuss a range of issues in this field, including methodological trends and and strengthening HTA capacity across the region (6). The network now includes twenty-nine HTA organizations from best practices, to build capacity among researchers and provide opportunities for regional and international collaboration.

With the aim of better understanding the characteristics of HTA systems and practices in this region, representatives from nine countries in Asia were invited to complete an online survey to describe their existing HTA processes. The countries covered in the analysis were: Bhutan, India, Indonesia, Japan, Malaysia, the Philippines, Singapore, Taiwan and Thailand. These countries are members of the HTAsiaLink network and have already adopted or are moving toward using HTA to inform healthcare policy. Each country varies considerably in terms of its level of economic development and health system characteristics as shown in Table 1. Representatives from other countries in the region were also invited to participate but did not provide responses. In the survey, representatives were asked to answer a series of open and closed-ended questions (questionnaire available in Supplement 1) about the processes in place in their respective countries with a focus on HTA evaluation and decisionmaking processes for inclusion of pharmaceutical products in government-sponsored benefits packages. A descriptive analysis of practices across countries was conducted and the key findings were organized around each core step of the HTA pathway (7): topic nomination and selection, technical assessment, evidence appraisal and decision making, dissemination of recommendations, and impact evaluation, as depicted in Figure 1.

**Table 1:** Country profiles

	Total population (2016)	Gross national income per capita (PPP$, 2013)	Total expenditure on health per capita (PPP$, 2014)	Total expenditure on health (% of GDP, 2014)	Out-of-pocket expenditure per capita (PPP$, 2015)	Out-of-pocket expenditure, as percentage of total health expenditure (%, 2014)	State/public expenditure informed by HTA, as percentage of total health expenditure (%)	Year national HTA unit/ committee was established
Bhutan	798,000	7,210	281	3.6	56.8	25.3		2009
India	1,324,171,000	5,350	267	4.7	154.7	62.4	30.0	2017
Indonesia	261,115,000	9,260	299	2.9	178.4	46.9		
Japan	127,749,000	37,630	3,727	10.2	577.0	13.9		2012
Malaysia	31,187,000	22,460	1,040	4.2	390.1	35.3	43.0	1996
Philippines	103,320,000	7,820	329	4.7	172.8	53.7	32.5	2018
Singapore	5,622,000	76,850	4,047	4.9	1161.5	54.8		2001^a^
Taiwan	23,516,000	42,040^b^	2,732^c^	6.3^c^		36.6^c^		2008
Thailand	68,864,000	13,510	600	4.1	71.8	11.9	80.0	2008

*Source*. WHO Global Health Observatory Indicators; http://apps.who.int/gho/data/node.imr.

a HTA has been used to inform national subsidy decisions in Singapore since 2001 when the Pharmacoeconomics and Drug Utilisation Unit (PEDU) was established. This group was superseded by the MOH Agency for Care Effectiveness (ACE) in 2015, which is the current national HTA agency.

b Data released from the Directorate General of Budget, Accounting and Statistics (DGBAS) of Executive Yuan, Taiwan (R.O.C.); https://eng.stat.gov.tw/public/Attachment/41019368L3E53FL6.pdf.

c Ministry of Health and Welfare 2017; https://www.mohw.gov.tw/dl-50681-9fc00da7-20fd-40d2-8024-fdd9281dcc35.html.

**Figure 1:**
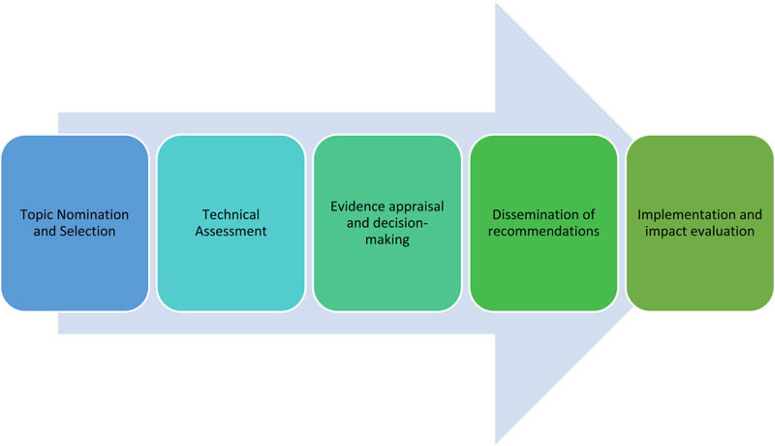
Core steps in the HTA pathway. Source: Adapted from Goodman CS (2014).

## Topic Nomination and Selection

All countries surveyed except for Taiwan (for the National Health Insurance new drug listing decisions) and Thailand have a national HTA agency or committee that plays a significant role in the topic nomination and/or selection processes (Table 2). In addition, among all countries, government bodies (e.g., national regulatory agencies, selection committees) and/or public healthcare institutions are integral to the process. However, there is some variation in how industry and private institutions participate in the process. Although some countries allow pharmaceutical companies to nominate topics for assessment, no country allows them to participate in the selection process. Similarly, patient representatives are less likely to inform which topics are selected, and are not typically included throughout the HTA pathway, except in Indonesia, the Philippines, and Thailand; however, many countries acknowledge that efforts to strengthen local patient engagement processes are needed over time, to ensure that HTA evaluations address the needs of all stakeholders.

**Table 2:** Stakeholders’ involvement in HTA topic nomination and selection process by country

	HTA agencies/committees		Other public institutions (hospitals, government bodies, regulatory agencies)		Health professional groups		Industry and private institutions		Patient advocacy groups, civil society, and general public	
	Nominate	Select	Nominate	Select	Nominate	Select	Nominate	Select	Nominate	Select
Bhutan		✔	✔							
India		✔	✔				✔^a^			
Indonesia		✔							✔	
Japan		✔								
Malaysia		✔	✔				✔			
Philippines		✔	✔		✔		✔		✔	
Singapore	✔	✔	✔		✔		✔^b^			
Taiwan^c^				✔			✔			
Thailand^d^			✔	✔	✔	✔	✔		✔	

a Industry and private institutes can nominate their topics to the HTA agency via a government body (center and state government departments/organizations). One such route is submitting the topic via National Healthcare Innovation Portal (http://nhinp.org/).

^b^Industry is periodically invited to suggest topics for HTA evaluation and subsidy consideration. Majority of the HTA topics are nominated by public healthcare institutions on an annual basis

^c^Referring to the process of the National Health Insurance new drug listing decisions.

^d^Referring to the process of the development of the National List of Essential Medicines.

Clear topic nomination and selection criteria are essential for identifying relevant HTA research topics that are aligned with local policies and priorities. Results showed that in all nine countries, HTA topics are selected using multiple criteria which are assessed using both quantitative and qualitative measures. Interventions which have significant clinical and/or economic impact or treat a condition which represents a high burden of disease in the local setting are more likely to be prioritized for evaluation. In addition, some countries (Bhutan, the Philippines, and Singapore) only select topics once a year whereas others have more frequent selection cycles, as shown in Table 3. In Thailand, for example, the Subcommittee for the Development of the National List of Essential Medicines (NLEM) meets on a monthly basis and can select topics for assessment during any of these meetings. However, we found that the number of HTA committee meetings held annually in each country is not correlated with the number of topics selected for evaluation; for example, in Singapore up to thirty topics are selected for assessment but the committee meets only two to three times a year.

**Table 3:** Country specific details on HTA topic selection, evaluation, and post-evaluation decision-making processes for pharmaceutical products

	Type of HTA for policy use (primary [P] or secondary [S] HTA^a^)	Topic selection		HTA evaluation	Post-evaluation decision making		
		Frequency of topic selection (per year)	Number of HTA topics selected for evaluation (per year)	Type of HTA producers (public [P] or private [Pr]^b^)	Frequency of decision making (per year)	Average time from submission to decision (months)	Name of decision-making committee/s
Bhutan	P, S	1		P	2–3	2–12	Ministry of Health
India	P, S	4–6		2–3	1–2	9–12	Ministry of Health and Family Welfare; National Health Authority (for National Insurance Scheme); State Health Departments; National Pharmaceutical Pricing Authority
Indonesia	P, S	2	4	8–10	N/A	N/A	HTA Committee
Japan	P, S			P, Pr	0.5	N/A	Central Social Insurance Medical Council (*Chuikyo*) of the Ministry of Health, Labour, and Welfare
Malaysia	S; P, S	3; 1 to 5	50; 5	P, Pr; P	3; 2	4; 5 to 8	Drug Selection Committee (Formulary); Health Technology Assessment & Clinical Practice Guideline Council (MaHTAS)
Philippines	P	1	6	P	24	12 (due to backlog)	Formulary Executive Council; Benefits Subcommittee of PhilHealth
Singapore	P, S	1	20–30	P	2–3	3–12	Ministry of Health Drug Advisory Committee
Taiwan	S	c	50+	P	6	12	Pharmaceutical Benefit Reimbursement Scheme (PBRS) Joint Committee meetings
Thailand	P, S	12 (maximum)	12–18	P	12 (maximum)	6–12	Subcommittee for National List of Essential Medicines

a Primary/Secondary: Primary data collection methods and secondary or integrative methods. Primary data methods involve collection of original data, such as clinical trials and observational studies. Integrative, or synthesis methods, involve combining data from existing sources (13).

b Examples of public producers: HTA agencies, academia, public research organizations; Private: consulting, pharmaceutical manufacturers (industry).

^c^ Manufacturers may submit listing applications to National Health Insurance Administrations (NHIA) at any time; HTA unit will accept all referrals from NHIA.

## Technical Assessment

The number of topics selected for assessment varies across the nine countries, ranging from two to more than fifty studies per year (Table 3), depending on each country’s technical capacity and assessment processes. For instance, Taiwan and Malaysia reported the highest number of HTA studies per year (fifty per year), possibly because both countries accept evidence submissions from pharmaceutical companies as part of their established HTA processes, reducing the need for in-house assessments. Countries relying only on the national HTA agency and/or local academic institutions to conduct HTA typically undertake fewer studies per year (ranging from two to three topics in Bhutan to twenty to thirty topics in Singapore) depending on the technical resources available. Representatives were not asked how long it takes to complete an assessment in each country; therefore, we were unable to determine whether this also influences the number of topics conducted in-house each year.

In terms of stakeholders involved, all countries reported that inform HTA evaluations. However, only three countries involve patients, and only one seeks inputs from the public.

Based on this analysis, countries can be categorized into two groups: (i) countries where decisions on topic selection and funding of a health technology and intervention are made by the same authority and (ii) countries where these decisions are made by different authorities. For the first group, the HTAs produced are usually policy relevant and may be more likely to be implemented than in the second group where the decisions on topic selection and HTA policy are made by different bodies. Nevertheless, there may be benefits in having separate bodies for making decisions on selecting topics and making funding decisions, because it promotes independence of the two decision-making processes, thereby reducing potential conflict of interest and ensuring that there is a balance of power in the system.

The countries reported differences in the mandates of their respective funding bodies, and it was noted that topics selected for assessment are largely dictated by local funding policies and national priorities. For example, vaccine and screening interventions are not covered under National Health Insurance (NHI) in Taiwan; therefore, only medicines are subjected to HTA to inform NHI funding decisions.

Finally, a majority of the countries surveyed (6) have developed HTA method guidelines to help guide local researchers through the HTA process and outline the methodological requirements and rigor required when they conduct HTAs. Among the countries surveyed, only two countries (Bhutan and the Philippines) do not have national HTA guidelines; however, they refer to international guidelines such as the International Decision Support Initiative (iDSI) Reference Case (8), ISPOR (9), and CHEERS checklist (10) when conducting HTA.

## Evidence Appraisal and Decision Making

All countries surveyed have quality assurance processes in place both before and after the HTA is completed, to strengthen the methodological rigor of their studies and ensure consistency across evaluations. For example, clinical stakeholder input is sought to ensure that the evidence available and base case analysis conducted are aligned with local practice. Further, internal review mechanisms and due diligence processes have been set up to ensure quality of evidence before the HTA is presented to decision makers. These mechanisms are critical, particularly in countries which accept evidence submissions from the industry. For example, the Center for Drug Evaluation (CDE) in Taiwan has developed in-house capacity to critically evaluate industry submissions used to inform funding decisions by the National Health Insurance Administration (NHIA). Other countries, such as Singapore and Thailand, also send in-house evaluations to independent reviewers, to validate the scientific rigor of their assessment, before it is used to inform funding decisions.

With regard to decision making, interestingly, the number of HTA studies conducted per year does not appear to be related to the time that HTA committees spend making funding decisions on the topics under consideration. For example, in Singapore, the Ministry of Health Drug Advisory Committee meets face-to-face relatively infrequently (two to three times per year), but funding decisions for more than ten topics are typically made at each meeting. Additional decisions for straightforward funding recommendations may be made by the Committee via email throughout the year. This practice raises questions on the optimal approach required for a timely and critical appraisal of HTA evaluations to inform decision making given the limited time that some committees have to convene and make decisions in each country.

## Dissemination of Recommendations

All national HTA agencies disseminate key findings of HTA studies and/or funding recommendations through their Web sites and peer-reviewed publications that are in English or in the local language. However, countries such as Singapore do not currently publish the full HTA evaluation report due to confidentiality considerations. In addition, countries surveyed share select evaluation methodologies and results at local, regional, or international scientific conferences. Some HTA agencies such as the Health Intervention and Technology Assessment Program (HITAP) in Thailand use other channels such as social media and have an active communication strategy in place to regularly distribute HTA studies or clinical findings to local professional associations and public healthcare institutions to influence clinical practice.

## Implementation and Impact

About half the countries (4) are involved in implementing HTA recommendations and conducting impact evaluations of specific interventions, but processes are still relatively inchoate and are not routinely applied to all topics. This reflects the global situation wherein evidence on the impact of HTA is limited (11). Only one HTA agency, MaHTAS in Malaysia, has so far established a formal feedback mechanism and measures the impact of HTA studies by surveying key stakeholders who requested HTAs. This process is based on the International Network of Agencies for Health Technology Assessment (INAHTA) framework for reporting on the impact of HTA reports (12). An analysis of the surveys from 1997 to 2016 showed that in ninety-six percent of cases, the HTA recommendations were accepted and were mostly used to inform provision of services (13).

## Conclusion

There is substantial variation in the HTA systems and practices reported among nine countries in the Asian region. This is perhaps unsurprising given the differences in the health systems, and economic development in terms of expenditure on health. However, despite these differences, there are similarities in terms of the processes and the role of HTA to inform funding decisions for pharmaceutical products in these countries.

This landscape analysis offers some lessons for countries that are either planning to reform their HTA system, or to establish HTA practices given limited technical and system capacity. In particular, our analysis highlights that it is crucial to find an optimum balance between the number of HTA studies conducted and the country’s technical capacity. If technical resources are limited, allowing industry submissions may be a way for more topics to be evaluated in a timely manner. In doing so, a process needs to be put in place to review submissions for their acceptability in the local context and determine whether additional resources would be needed to manage engagement with industry, critique submissions, and support pharmaceutical companies during the submission process. The case of Australia, not covered in this analysis, provides an example of such a process whereby the Pharmaceutical Benefits Advisory Committee (PBAC) operates by having evaluators assess industry submissions, rather than having evaluators conduct evaluations themselves. Another option to maximize limited resources could be to explore the feasibility of adapting HTA findings from other regions to the local context.

Capacity constraints and governance principles should be considered while setting up HTA processes in line with the local context. Countries may weigh the benefits and challenges of having a single decision-making authority to select topics and make funding decisions, as there may be a conflict of interest, compared to having separate decision makers for each of these functions. In terms of the scope of HTA, it may be impossible to adopt HTA to inform funding and clinical practice decisions for all health interventions given the limited resources and capacity in Asia. Until resources increase in countries with constrained resources, focusing on priority interventions could be a feasible option to ensure that HTA evaluations are targeted at research topics that will be most impactful and will maximize benefits to patients within the finite resources available. This also applies to the appraisal process, whereby the frequency and duration of committee meetings may influence the number of topics that can feasibly be considered each year by decision makers. Hence a balance between the number of committee meetings and number of submissions reviewed per meeting is required.

Our findings illustrate the importance of learning and reflecting on current practices and systems for an evolving HTA landscape in Asia. Collaborations among HTA agencies in the region as well as with global partners can address the capacity gaps. However, the heterogeneity in practices and healthcare systems suggests that there are a number of challenges for implementing a single HTA system in the region, an approach that has been proposed in Europe (14), to increase efficiency and address the limited capacity for HTA in the region. Further research in this area is needed to explore the feasibility of establishing a harmonized HTA system in Asia. In addition, other countries within the region, particularly those which already have established HTA processes, such as the Republic of Korea and the People’s Republic of China, which were not included in our analysis, should also be studied to ensure that any future recommendations for a single HTA system take into account all of the processes already in place across the region.

## Notes

Supplementary Material. The supplementary material for this article can be found at https://doi.org/10.1017/S0266462319000667
