# Supplementary material for: Landscape analysis of health technology assessment (HTA): systems and practices in Asia
Source: Int J Sci Res (Raipur). 2019 Jun 9;35(6):416–21. doi: 10.1017/S0266462319000667 (PMC7722344; doi:10.1017/S0266462319000667)
Supplement: Supplementary file 1 [file IJTAHC-35-06-416-s001.pdf]

HTA Country Survey

Start of Block: Introduction

Q1.1 Institutionalizing HTA is powerful in guiding policy and clinical practice towards best possible cost and health outcomes; however, this is done differently across countries and contexts. This study is an effort to understand and appreciate the way HTA processes are integrated in the policy making processes across countries in the Asia-Pacific. Along with understanding the technical evaluation process, this study aims to learn about the ecosystem supporting HTA evaluations, the pre and post evaluation processes and the way its integrated in the country's health systems.
We are glad to inform that representatives from HTA agencies and experts from around 10 countries in the region have agreed to contribute to this survey. We would be performing a comparative analysis and circulating the results across for feedback to ensure we accurately represent your input. Thank you for your time and contribution to this learning effort.
We recommend that you finish the entire questionnaire in one sitting. However if you are unable to do so, please close the browser and click on the survey link again (using the same internet browser) and you can continue with the questionnaire from where you have last left off.

For any queries or technical issues relating to the questionnaire, please contact us at hiper@nus.edu.sg
Thank you!

Q1.2 Which country's HTA agency are you currently representing?

____

End of Block: Introduction

Start of Block: HTA system

Q2.1 **Section A: Overview of the HTA system in Your Country**

Q2.2 Which are the features of your healthcare financing system that require evidence developed through HTA (check all that applies)?

- ☐ Public-funded schemes at national level (1)
- ☐ Public-funded schemes at state level (2)
- ☐ Social insurance scheme (government- and/or employer-funded) (4)
- ☐ Private insurance scheme (3)
- ☐ Patient's out-of-pocket expenditure (5)
- ☐ Others (6) ____

Q2.3 What proportion of healthcare financing expenditure is represented by schemes supported by HTA-informed processes? Also, how significant are these schemes among your country's public health programs.

____

____

____

____

____

Q2.4 Who are the decision makers using HTA evidence in policy makers?

____

____

____

____

____

Q2.5 In which year did decision makers begin using HTA evidence regularly?

____

Q2.6 Which other stakeholders are involved in the deliberation process in your country (check all that applies)?

- ▢ Clinical working groups (1)
- ▢ Patient groups (2)
- ▢ Manufacturers (3)
- ▢ Academia (4)
- ▢ Healthcare institutions (5)
- ▢ Others (6) ____
- ▢ None of the above (7)

Q2.7 Which party or parties in your country produce the HTA evidence (check all that applies)?

- [ ] National HTA agency (1)
- [ ] Academics (2)
- [ ] Consulting firms (3)
- [ ] Manufacturers (4)
- [ ] Others (5) ____

Q2.8 Is HTA evidence recommended or requested by legislation?

- ○ Yes (1)
- ○ No (2)
- ○ Unsure (3)

Display This Question:

If Is HTA evidence recommended or requested by legislation? = Yes

Q2.9 Please name the legislation.

____

End of Block: HTA system

Start of Block: Topic selection

Q3.1 **Section B: HTA Topic Selection in Your Country**

Q3.2 Which group(s) select topics for evaluation (check all that applies)?

- [ ] HTA agency (1)
- [ ] Manufacturers (4)
- [ ] Public healthcare institutions (2)
- [ ] Private health institutions (7)
- [ ] Patient advocacy groups (3)
- [ ] Others (6) ____

Q3.3 Which group(s) are allowed to submit requests for HTA evaluations (check all that applies)?

- [ ] Manufacturers (1)
- [ ] Public health institutions (2)
- [ ] Private health institutions (3)
- [ ] Patient advocacy groups (4)
- [ ] Others (5) ____

Q3.4 On average, how many times are topics selected in a year?

- ☐ Pharmaceutical products (1) ________________
- ☐ Medical devices/diagnostics (2) ________________
- ☐ Medical technology (3) ________________
- ☐ Gene therapy (4) ________________
- ☐ Others (5) ________________

Q3.5 On average, how many topics are selected in a year by the selection committee?

- ☐ Pharmaceutical products (1) ________________
- ☐ Medical devices/diagnostics (2) ________________
- ☐ Medical technology (3) ________________
- ☐ Gene therapy (4) ________________
- ☐ Others (5) ________________

Q3.6 Who makes the final decisions for topic selection in your country?

____

Q3.7 What would be the criteria used for topic selection by the agency/evaluation team?

Q3.8 Are there any topics outside the remit of HTA evaluations, e.g., vaccinations?

- Yes (1)
- No (2)
- Unsure (3)

Display This Question:

If Are there any topics outside the remit of HTA evaluations, e.g., vaccinations? = Yes

Q3.9 Please elaborate why are these topics outside the remit of HTA evaluations in your country.

End of Block: Topic selection

Start of Block: HTA evaluation process

Q4.1 **Section C: HTA Evaluation Process in Your Country**

Q4.2 What types of HTA evaluations are produced for policy use (check all that applies)?

- ▢ Locally-produced Full HTA evaluation (1)
- ▢ Locally-produced expedited HTA evaluation (2)
- ▢ Full HTA evaluation based on international data (3)
- ▢ Expedited HTA evaluation based on international data (4)
- ▢ Others (5) ____

Q4.3 Are there any processes or mechanisms that allow other stakeholders' involvement during evaluation to ensure its relevance?

- ◯ Yes (1)
- ◯ No (2)
- ◯ Unsure (3)

Display This Question:

If Are there any processes or mechanisms that allow other stakeholders' involvement during evaluatio... = Yes

Q4.4 Who are these other stakeholders (check all that applies)?

- ☐ Clinical working groups (1)
- ☐ Patient advocacy groups (2)
- ☐ Academias (3)
- ☐ Decision-makers (4)
- ☐ Manufacturers (5)
- ☐ General public (6)
- ☐ Others (7) ____

Display This Question:

If Are there any processes or mechanisms that allow other stakeholders' involvement during evaluatio... = Yes

Q4.5 At what stage(s) of the HTA evaluations are these stakeholders involved (check all that applies)?

- ☐ Design of HTA evaluation plan (e.g. appropriate model choice, treatment pathways) (1)
- ☐ Fine-tuning of the HTA analysis (e.g. providing more accurate clinical data) (2)
- ☐ Recommendations (3)
- ☐ Others (4) ____

Q4.6 Do you have country-specific evaluation guidelines?

- ○ Yes (1)
- ○ No (2)
- ○ Unsure (3)

Display This Question:

If Do you have country-specific evaluation guidelines? = Yes

Q4.7 When was the first version established?

____

Display This Question:

If Do you have country-specific evaluation guidelines? = Yes

Q4.8 Who established the very first guidelines?

____

Display This Question:

If Do you have country-specific evaluation guidelines? = Yes

Q4.9 How many versions of the guidelines have been published since the first version?

____

Display This Question:

If Do you have country-specific evaluation guidelines? = No

Or Do you have country-specific evaluation guidelines? = Unsure

Q4.10 Which existing guidelines does the agency commonly refer to for HTA process and evaluations (check all that applies)?

- ☐ CHEERS (1)
- ☐ ISPOR (2)
- ☐ Others (3) ____

Q4.11 **The following questions are in reference to the country-specific or reference guidelines that your country is currently using (e.g. CHEERS, ISPOR).**

Q4.12 What perspective/s does the evaluation team take in the economic evaluation of health technologies (check all that applies)?

- ☐ Patient (1)
- ☐ Healthcare (2)
- ☐ Societal (3)
- ☐ Others (4) ____

Q4.13 What are the time horizons commonly considered in evaluation?

____

____

____

____

____

Q4.14 When would the below types of economic evaluations be preferred in your country, and why?

- ☐ Cost-minimisation (1) ________________
- ☐ Cost- benefit (2) ________________
- ☐ Cost-effectiveness (3) ________________
- ☐ Cost-utility (4) ________________

Q4.15 What would be the acceptable sources of clinical data in your country (e.g. disease registries, hospital databases) (check all that applies)?

- ☐ Disease registries (1)
- ☐ Hospital administrative databases (2)
- ☐ Literature reviews (3)
- ☐ International evidence (4)
- ☐ Clinical trials data (5)
- ☐ Others (6) ________________

Q4.16 If real world evidence are used, are there guidelines for using them?

____

____

____

____

____

Q4.17 What would be acceptable levels of clinical evidence (i.e. hierarchy of study design) (check all that applies)?

- ▢ Systematic reviews/ meta analysis (1)
- ▢ Randomised controlled trials (RCTs) (2)
- ▢ Non-RCT (3)
- ▢ Cohort studies (4)
- ▢ Case-control studies (5)
- ▢ Case reports (6)
- ▢ Others (7) ____

Q4.18 What are the preferred measures of effects in your country (check all that applies)?

- ☐ Surrogate health outcomes (e.g. clinical indicators-HbA1c) (1)
- ☐ Final outcomes (e.g. deaths averted) (2)
- ☐ Utilities-DALYs (3)
- ☐ Utilities-QALYs (4)
- ☐ Patient reported outcomes measurements (e.g. pain level) (5)
- ☐ Others (6) ____

Q4.19 What would be the common sources of pricing information used in estimating direct medical cost (check all that applies)?

- ☐ Hospital administration data/bill pricing (1)
- ☐ International pricing (2)
- ☐ Current procurement price (3)
- ☐ Manufacturers new offer price (4)
- ☐ Others (5) ____

Q4.20 What would be the common sources of pricing information used in estimating direct non-medical cost (e.g. transportation cost to hospital) (check all that applies)?

- ☐ Patient survey (1)
- ☐ Estimation by proxy (e.g. distance between home and hospital) (2)
- ☐ Others (3) ____

Q4.21 What would be the common sources of pricing information used in estimating indirect cost (e.g. productivity losses) (check all that applies)?

- ☐ Patient survey (1)
- ☐ National average/median income (2)
- ☐ Others (3) ____

Q4.22 Is discounting recommended?

- ○ Yes (1)
- ○ No (2)
- ○ Unsure (3)

Display This Question:

If Is discounting recommended? = Yes

Q4.23 Are the discount rates different for the health outcomes and costs? Why or why not?

____

Display This Question:

If Is discounting recommended? = Yes

Q4.24 What is the discount rate(s) used?

- ☐ For cost: (1) ____
- ☐ For effect: (2) ____

Q4.25 What would be the recommendations for handling uncertainty in your country?

____

____

____

____

____

Q4.26 What is the cost-effectiveness threshold/ceiling threshold used for decision making after evaluation?

____

Q4.27 Are there different WTP thresholds used for different technologies/conditions?

- ○ Yes (1)
- ○ No (2)
- ○ Unsure (3)

Display This Question:

If Are there different WTP thresholds used for different technologies/conditions? = Yes

Q4.28 What are the respective WTP thresholds for these different technologies/conditions?

____

____

____

____

____

Q4.29 Are there recommendations or guidelines for budget impact analysis (BIA) in your country?

- ◯ Yes (1)
- ◯ No (2)
- ◯ Unsure (3)

Display This Question:

If Are there recommendations or guidelines for budget impact analysis (BIA) in your country? = Yes

Q4.30 What is the preferred perspective of the BIA?

____

Display This Question:

If Are there recommendations or guidelines for budget impact analysis (BIA) in your country? = Yes

Q4.31 What are the preferred time horizons for BIA?

____

Q4.32 Would there be some quality assurance process for the HTA report?

○ Yes (1)

○ No (2)

○ Unsure (3)

Display This Question:

If Would there be some quality assurance process for the HTA report? = Yes

Q4.33 Please elaborate on these quality assurance processes.

____

____

____

____

____

End of Block: HTA evaluation process

Start of Block: Post-HTA

Q5.1 **Section D: Post-HTA Decision-Making Processes in Your Country**

Q5.2 How many times in a year does the decision making committee meet for decision making?

____

Q5.3 What would be the average time needed for decision making between the submission of HTA report and final decision?

____

Q5.4 How would the decision committee account for other social or ethical considerations?

____

____

____

____

____

Q5.5 Are there mechanisms in place to allow for appeal post decision making?

- Yes (1)
- No (2)
- Unsure (3)

Display This Question:

If Are there mechanisms in place to allow for appeal post decision making? = Yes

Q5.6 What are the criteria for appeal following decision making?

____

____

____

____

____

Q5.7 Are price negotiations carried out in your country?

- ◯ Yes (1)
- ◯ No (2)
- ◯ Unsure (3)

Display This Question:

If Are price negotiations carried out in your country? = Yes

Q5.8 How are these price negotiations carried out?

____

____

____

____

____

Q5.9 What are existing channels for disseminating recommendations to various stakeholders (e.g. clinicians, manufacturers, the general public) (check all that applies)?

- ▢ Official government websites (1)
- ▢ Online and offline media sources (Newspaper, radio) (2)
- ▢ Report disseminated to various healthcare institutions (3)
- ▢ Medical associations (4)
- ▢ Roadshows (5)
- ▢ Others (6) ____

Q5.10 What are the mechanisms used to encourage greater dissemination and implementation of HTA decisions?

____

____

____

____

____

Q5.11 How is the implementation of HTA findings monitored following the evaluation?

____

____

____

____

____

End of Block: Post-HTA
